# Children's perspectives and experiences of the COVID‐19 pandemic and UK public health measures

**DOI:** 10.1111/hex.13350

**Published:** 2021-09-08

**Authors:** Jill Thompson, Grace Spencer, Penny Curtis

**Affiliations:** ^1^ Health Sciences School, Division of Nursing and Midwifery The University of Sheffield Sheffield UK; ^2^ School of Education and Social Care Anglia Ruskin University Cambridge UK

**Keywords:** children, COVID‐19, public health, qualitative research

## Abstract

**Background:**

The COVID‐19 pandemic has had profound impacts on how we live our lives; yet, the implications for children and the effects on children's everyday lives have been relatively underacknowledged. Understanding children's views on COVID‐19 and related restrictions on their lives provides an important opportunity to understand how children have responded to the pandemic, including the impacts on their social and emotional well‐being.

**Objective:**

This study explored the experiences and perspectives of children in relation to the COVID‐19 pandemic and related restrictions on everyday life.

**Method:**

A qualitative study using semistructured online interviews with participatory drawings was undertaken between May and July 2020. Eighteen children from England and Wales, aged 7–11 years, participated in interviews.

**Findings:**

Themes included children's reflections on (1) COVID‐19 as a deadly contagion; (2) fears and sadness and (3) social responsibility and opportunities to respond positively.

**Conclusions:**

Young children offer insightful reflections on their experiences during the first wave of the COVID‐19 pandemic in the United Kingdom. Children and young people's perspectives must be considered in future public health discourse.

**Patient or Public Contribution:**

This work was informed by conversations with my own three primary school‐aged children and the children of friends. A formal Patient and Public Involvement (PPI) group was not established due to the speed with which the project was undertaken. Any future research in this area would benefit from formal PPI in the design, methods and questions.

## 1 INTRODUCTION

Across the globe, the COVID‐19 pandemic has profoundly affected the ways in which we all live our lives. In the United Kingdom, and other many other countries, much of the political and public health response has been adult‐centric, with the roles and perspectives of children on the pandemic and how best to respond to this less well considered. Rather, children and young people have been differentially positioned as either ‘resilient’, able to simply and unproblematically ‘bounce back’ from the pandemic, or as ‘deviant’ and largely culpable for (ongoing) transmission of the virus because of their ‘failure’ to adhere to public health guidance and maintain social distancing. The relative absence of children's perspectives within the development of public health strategies not only serves to downplay the impacts that COVID‐19 has, and continues to have, on young lives but also runs the risk of failing to engage sensitively and meaningfully with the different ways in which children understand and respond to the pandemic, including what this might mean for their engagement with public health advice.

Drawing on a qualitative study with 18 children in England and Wales, this paper explores children's understandings of, and reflections on, COVID‐19 and the related political and public health responses to the pandemic. The findings reported here highlight children's thoughts and fears about the scale and impacts of the pandemic, and explore how they have taken up key public health messages to protect themselves and others. The findings also highlight the importance of reframing health promotion messages to better capture children's own perspectives on, and roles within, public health responses to the pandemic. We begin by foregrounding children's contributions to health promotion and the value of attending to these perspectives within the development of public health programmes, before detailing the study and its main findings.

## 2 CHILDREN'S PERSPECTIVES AND THE DEVELOPMENT OF PUBLIC HEALTH PRIORITIES

The development of public health priorities is usually underpinned by an assessment of epidemiological measures including morbidity and mortality rates, and related indicators of health practices or individual risk behaviours (e.g., smoking, exercise, diet, alcohol use). Dominant forms of health promotion thus aim to educate people about the risks tied to harmful health practices and encourage them to adhere to official health advice. These traditional forms of health education typically depict an uncritical, compliant health consumer who engages in proscribed behaviour change, often without due regard to the (differing) meanings attached to health and related health practices.^1^, ^2^

Health education with children has followed these models, adopting a didactic approach to providing formal health knowledge. Children are expected (uncritically) to follow instructions and modify their behaviours accordingly.^3^ These approaches position children as ‘empty vessels’ to be ‘filled’ with ‘appropriate’ health information to make expert‐defined ‘right’ choices.^1^, ^4^ Children's ‘failure’ to follow such advice is often attributed to assumptions about their lack of knowledge, ‘immaturity’ and inabilities to know what is best for them and their health.^2^ Because of this, adult frames of reference often define the forms of health promotion deemed most appropriate for children. Not only do such approaches downplay children and young people's own understandings of health and health promotion^1^, ^5^ but they also neglect the relevance of the context in which health is experienced and enacted.

Critiques of adult‐centric health promotion agendas have emerged in recent times.^5^, ^6^ Drawing on contributions to the sociology of childhood,^7^, ^8^ these perspectives foreground children and young people's capacities and capabilities with respect to their health‐related decision‐making and health practices. In line with Article 12 of the United Nations (UN) Convention on the Rights of the Child,^9^ these contributions underscore the importance of capturing children's own views on health and the subsequent grounding of health promotion within the lived realities of young lives. Indeed, in recent times, there have been concerted efforts to engage children and their perspectives in health care planning as part of broader Patient and Public Involvement (PPI) initiatives in health care, especially within the UK context.^10^, ^11^

Despite the increasing recognition of the importance of eliciting children and young people's views on topical health concerns and public health priorities, there has been a notable absence and failure to engage with children's perspectives on COVID‐19 within political and public health decision‐making (The New Zealand Prime Minister, Jacinda Arden, offers an exception to this and held briefings and conversations with children at the beginning of the pandemic^12^). The impacts of COVID‐19 on children's present and future lives in terms of their socio‐emotional and mental health, their education and future career prospects are beginning to emerge.^13^, ^14^, ^15^ Indeed, children (increasingly termed the COVID generation) are likely to experience the impacts and consequences of the pandemic for many years to come and as education and career prospects are compromised and reconsidered. To address the absence of young perspectives in the pandemic discourse, we undertook a qualitative study with young children in England and Wales during the first wave of the pandemic to better understand their experiences of COVID‐19 and how these perspectives may help to inform future health promotion strategies.

## 3 METHODS

A qualitative study with 18 children (9 boys and 11 girls) aged 7–11 years in England and Wales was conducted between May and July 2020. During this period, most children in England and Wales were experiencing a prolonged period of home schooling as part of a national lockdown, with schools having been closed since late March 2020. None of the children involved in the study were currently attending school in person due to the national lockdown.

### 3.1 Recruitment and sampling

The study was advertised on two parent support groups on the social media platform, Facebook. Interested parents were encouraged to contact the researcher, J. T., via email. Following contact, an information sheet and consent form were emailed to parents. All those who expressed an interest in the study were interviewed.

### 3.2 Data collection

Twelve semistructured online interviews with participatory drawings were conducted with 18 participants (six paired interviews were conducted, with siblings being interviewed at the same time). This method has been successfully used in other research exploring children's understandings of health.^16^, ^17^, ^18^ Drawings encourage children to share their thoughts to some initial questions in a visual format. In practice, some children in this study were more enthusiastic about drawing than others, with some children simply preferring to talk about their experiences. Difficulties were experienced, for example, in angling cameras for the researcher to see drawings. Because of this, the researcher asked the children to describe what they were drawing and to hold up their drawings at different points during the interview.

To aid discussions, a flexible interview guide was developed and focused on the following broad topic areas: children's understandings of COVID‐19 and its transmission (e.g., ‘Have you heard of COVID‐19 or the coronavirus’?, ‘What do you think it is’?, ‘How does it get into people's bodies’?); symptoms of COVID‐19 (e.g., How do people feel if they have COVID‐19?); those most at risk from the virus (e.g., ‘Do you think that there are some people who are more likely to get poorly with COVID‐19?’); and children's everyday lives during lockdown (e.g., ‘Tell me what you did last week’?, ‘What are some of the things that you like about lockdown’?, ‘What are some of the things that you don't like about lockdown’?).

Interviews were conducted by J. T. and lasted between 30 min and 1 h. Some parents remained present during the interviews, with some actively contributing to discussions (the implications and impacts of this are discussed later in the paper). Others left their children, enabling them to talk privately to the researcher. All interviews were audio‐recorded with participants’ consent (and their parents’). Following the interviews, parents were asked to email photographs of their children's drawings to J. T.

### 3.3 Data analysis

Interviews were transcribed verbatim by a professional transcription company. Transcripts were fully anonymized, and participants were given pseudonyms (some were the child's own choice) and thematically analysed.^19^ First, transcripts were (re)read and descriptive codes were attached to the data by J. T. Codes were then discussed and cross‐checked with G. S. and P. C. to enable alternative ‘readings’ of the data. Descriptive codes were then scrutinized, refined and grouped by J. T. and P. C. to identify topical categories emerging from the data. Categories were then compared within and across transcripts to identify synergies and departures. Topical categories were developed into core thematic areas. The drawings were analysed alongside the children's transcripts and to reflect the specific issues that the children were discussing at the time.

### 3.4 Ethical considerations

Ethical approval for the study was received from the Health Sciences School ethics committee at The University of Sheffield. Verbal consent from parents and assent from children were taken at the time of the interview. Participants and their parents were advised about the aims of the study, the nature of their involvement and what would happen with their data in line with data protection requirements. Participants were free to withdraw from the interview at any time and without needing to give a reason.

## 4 FINDINGS

Our analysis revealed three key themes that captured the ways in which children spoke about and experienced the pandemic, namely, (1) A deadly global contagion, (2) Fears and sadness and (3) Social responsibility and opportunities to respond positively.

### 4.1 A deadly global contagion

**Figure 1:**
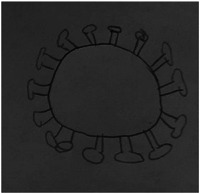
Rose, aged 11

Participants provided clear verbal descriptions and drawings of what they thought the COVID‐19 virus looked like and how this was spread, which often reflected mainstream media images of the virus, as seen in Rose's drawing (Figure 1). When probed about their drawings, children confirmed that they had seen similar images of the virus in the news.

**Figure 2:**
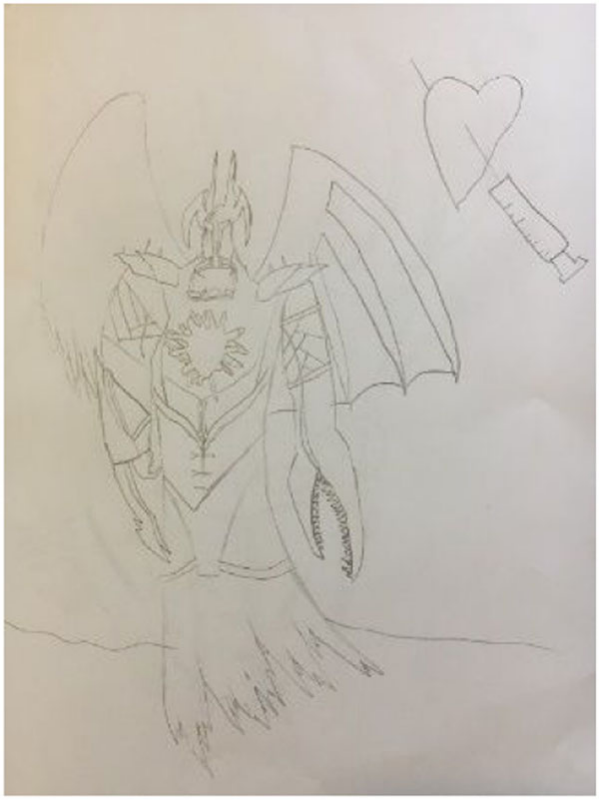
Ray, aged 10

Other children, such as Ray (aged 10), likened the virus to a monster killing people and thus something to be defeated (Figure 2), whilst Ben's (aged 9) description hinted at the notion of contagion as he described coronavirus as an invisible yellow gas surrounding people:

*…sometimes I imagine people kind of had the invisible gas around them. That in my head is yellow, the gas is sometimes imagined 2 metres spread and that's the Coronavirus*. (Ben, aged 9)

*I'm drawing a monster and it has the Coronavirus on different parts of it because Coronavirus is a monster…Because it's killing loads of people, it's trying to be stopped and kept captive*. (Ray, aged 10)

**Figure 3:**
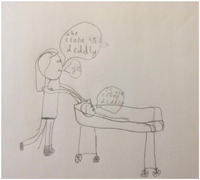
Abigail, aged 8

**Figure 4:**
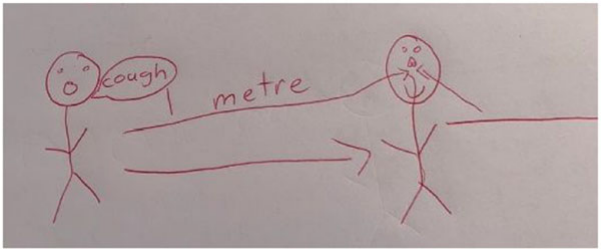
Sophie, aged 9

When asked what COVID‐19 does to people, some children talked about the physical impacts and symptoms of the virus including a cough and a high temperature. As Ben's description above highlights, participants often talked about the deadly nature of COVID‐19, often sharing their concerns and fears about the virus harming others. Abigail's (aged 8) drawing of the impacts of COVID‐19 (Figure 3), for example, illustrates a person laying in a bed asking, ‘Is the [Corona] deadly’? The person standing above the bed replies, ‘Yes. The [Corona] is deadly’:

Children's discussions about the harmful physical effects of COVID‐19 were further evident in their accounts of how the virus was transmitted. Children described how the virus could be spread from one body to another, although there was considerable variability in children's understandings of how the virus may enter the body. When Sophie described her drawing (Figure 4), she talked about how germs entered the body through the nose and mouth and because of poor hygiene on the part of the individual. Others suggested that COVID‐19 might enter the body through the skin or mouth and nose.

* …they've kept touching the bar and then they didn't wash their hands and then they touched their mouth and their nose and their face and then he's coughed from 1 metre, not 2 metres, and not covering with his elbow and its gone down into his body and gets the germs into their nose*. (Sophie, aged 9)

*I think there's like these tiny cracks in your skin and they can just travel through and get into your body*. (Emma, aged 8)

*It could get into cuts and through your mouth and nose*. (Oscar, aged 9)

These accounts can be read as evidence of children's uptake of key public health messages in response to COVID‐19 and about how to mitigate personal risks and the risk presented to others. For example, dominant health advice stresses the importance of adhering to handwashing and observing guidance on social distancing, as echoed in Albie's and Sophie's (both aged 9) responses:

*Social distancing, wear masks, try not to go out too much and don't meet up with people*.

*Social distance, stay in the house and only the mums and dads are allowed to go to the shops and only go out for walks, not going to big group meetings and stuff*.

As well as displaying their knowledge of public health messages, children also demonstrated their political and global astuteness as they discussed the origins of the virus (locating this in China) and the global spread. Other children reflected carefully and critically on the different political responses to the pandemic and in doing so, criticized in‐country governments (e.g., the UK government) for poor handling of the pandemic, which contributed to the virus spreading:

*It's [COVID‐19] come from China because they were eating bats…Because the bats, I think the bats had a disease that's spreadable that humans can get and because Chinese people were eating them, they were getting the disease and passing it on to loads of people and they were going to different places in the world*. (Rose, aged 11)

*…why is the government making such bad decisions about it? I mean, if Boris Johnson already has Coronavirus, government you are doing a beeping job! … They're doing a rubbish job—New Zealand's doing great. I mean the Coronavirus has gone in New Zealand*. (Ray, aged 10)

### 4.2 Sadness and fears

Children's discussions of the virus often signalled their deep fears and anxieties about the pandemic. Fears were triggered by a sense of the unknown, and especially not knowing when life would get back to normal. For instance, when asked if anything worried Oscar (aged 9) about the pandemic, he replied, ‘About getting it, and it not stopping and having to live like this forever’. As previously noted, talk of death and the scale of deaths was commonplace in children's reflections:

*I think it's really, a really terrible virus. And lots and lots of people are dying, it's really, really sad. And I'm not really, really, really used to it [death]*. (Thomas, aged 7)

Some children shared their anxieties about their own risk to COVID‐19 and impacts on their health. Rose (aged 11) reported, ‘It makes me feel a bit scared that if I was to get it, I could get really ill’. However, more often, children reported their concerns for other more vulnerable people, in particular, family and friends:

*Maria: I don't like it that my friends could be poorly and dead if they're poorly of the Coronavirus*

*Interviewer: Do you feel worried about Coronavirus? What worries you?*

*Ben: I worry that it's going to start affecting people in my family. And my friends and maybe when I get back, maybe when the Coronavirus ends, I won't have all the people that my friends—I might not have all the people that I love in my family*. (Ben aged 9, Maria aged 7; siblings, interview 10)

Reports about losing the people they love highlighted some of the worrying and unintended impacts that dominant public health narratives may have on children's mental well‐being. Along with concerns about virus risk for family members and friends, children's accounts also reflected their sadness about the things that they missed due to COVID‐19 restrictions. They described how they longed for physical contact with others, along with missing out on sports and extracurricular activities or key school events, such as their final year of primary school and residential trips. For example, Rose spoke about her sadness following the abrupt end to her primary education and how she missed out on important opportunities that would aid her transition to secondary school:

*When I last went to school, I was really sad that we were going to have to go and then when I didn't know that school was going to close, I was really excited to have a leavers’ assembly and leavers’ jumpers and my SATS. But obviously when I found out that I couldn't have leavers’ jumpers or assembly, I was really upset. I'm not really excited that much to go to high school because it's a bit too early, I haven't done SATS or anything*. (Rose, aged 11)

Children's reflections on the things that they missed and aspects of previous life were clearly marked with sadness. Thomas (aged 7) talked about a recent bike ride with a friend and how he had wanted to hug him, but could not, stating, ‘[it] makes me feel seriously devastated’, whereas Maria (aged 7) described current life as:

*Terrible, because I can't see my friends, or my teacher. Because I am really sad, I can't see my bestest friend. I can't see her when the Coronavirus has stopped either—she's moved to another house and school*.

Missing physical contact with others such as hugs with friends also featured in participants’ accounts. Children were clearly grappling with what they wanted to do and what they were being told they could do.

*Abigail: You didn't have to social distance and you could have people that could touch you. Or I want to touch something and you're allowed to touch and you're like ‘oh that's my friend, can we go and meet them?' And then you hear ‘no we're not allowed to do that’*.

*Laura: Yeah, so all them regular things that we used to do have turned into ‘try not to touch anything when you want’*.

*Interviewer: How does that make you feel?*

*Laura: Well sometimes sad because I can't like hug my cousins and my friends and I want to, and then other times I think, yeah, I understand*.

### 4.3 Children's positivity and social responsibility

Despite sharing their anxieties, participants also highlighted some of the more positive aspects of the pandemic and the opportunities that it could afford. Some children talked positively about their broader social responsibility and, when doing so, pointed to examples of the apparent lack of responsibility of others. During these discussions, participants appeared to align with dominant responsibilization narratives in relation to older children and young people as they described them as ‘naughty’ for not observing social distancing rules.

*…I've been seeing like lots of teenagers and naughty people just going like high fives, cuddles—what are they doing? If they want this to end, just stop touching each other! [W]henever I see people touching each other, I just get really upset, cos it's kind of getting things worse*. (Eva, aged 9)

Others spoke more positively and reflected their sense of pride at being part of a larger movement and time in history.

*I actually feel excited and curious because this time on lockdown will be remembered for thousands and thousands of years*. (Rose, aged 11)

*[I]t's sort of nice to be, like, living through something that might not happen again, people are going to, like, write about it in history books and so people of the future will learn about it*. (Alice, aged 11)

*I'm happy that we're all safe and that we're all working together to stop it …I just feel so proud that everyone's working together…and we're all doing our bit*. (Eva, ages 9)

Many participants spoke fondly about lockdown, including opportunities to spend more time with family. When asked about the things that made her happy during this time, Laura (aged 9) said:

*Spending more time with my mum and dad because at school we are away from them for at least 7hours because we have to get collected by someone else, so it's nice to get a full day with them*.

Children also described the benefits of spending more time outdoors, playing in their gardens, riding their bikes or taking walks with their family, often discovering new outdoor spaces. There was a clear sense that many children enjoyed the freedoms afforded to them by not being in school and the opportunities to fill their own time.

*I like that we're still allowed to walk outside, and like have some fresh air and explore new places*. (Eva, aged 9)

*We have quite a big garden and we own part of a field and so we've been going outside a lot, which is really fun*. (Alice, aged 11)

Emma (aged 8) highlighted the greater sense of freedom that some children enjoyed during the lockdown and school closure period:

*Interviewer: And is there anything that you enjoy about being at home now?*

*Emma: Maybe that we get to do more things than we did at school. I've done some baking and we have played outside a lot more. And we've started doing a lot of drawing*.

Discussions with children also revealed how some were engaging with new and emerging technologies, which helped them maintain a sense of social connectedness with their friends and play remotely and synchronously. Rose (aged 10) discussed using mobile phones, ‘so we can play a game and call at the same time’. Similarly, Ben (aged 9) talked about playing interactive games with friends on his Nintendo Switch and using video conferencing and other new interactive apps to play live games with his friends. Thomas shared how he was using video conferencing and interactive games to connect with friends in virtual ‘worlds’:

*I've been talking to a lot of people from Zoom. Even on Minecraft I manage to friend people so they can play in my worlds*. (Thomas, aged 7)

These accounts, and others, point to the ways in which some children were actively seeking out opportunities for alternative ways to play ‘freely’, for example, by inviting friends to play within their own virtual worlds, where they may have felt a greater sense of control and the opportunity to create their own rules. Indeed, these children offered frequent examples of how they had embraced the changes and harnessed their agency to reflect on what they could do, rather than focus on what they could not.

## 5 DISCUSSION

The findings reported in this study illustrate that young children are knowledgeable about the COVID‐19 pandemic, its transmission, the risks and related public health harm reduction strategies. Such knowledge is evident despite the obvious omission of children's perspectives in the political and public health discourse surrounding COVID‐19. The British Science Association^20^ recently warned of the possibility that young people may feel alienated and disassociate with pandemic guidance if they are not included in the conversations. Our research highlights the important insights that children can bring to the policy‐making arena and we would strongly recommend that children are engaged in any future discussions that reflect on the UK's approach to managing the pandemic and also discussions about future global challenges that will have direct impact on children's lives.

Such calls to involve children and young people in matters that impact significantly on their daily lives, including health research and policy, are not new. Numerous authors have pointed repeatedly to the important insights that children can offer regarding their own health and well‐being. However, over 30 years since its inception, Article 12 of the UNCRC is still not enforceable in UK courts.^21^, ^22^ Clearly, much work remains to be undertaken to ensure that children's views on all aspects of their lives are given the prominence that they deserve.

Throughout the COVID‐19 pandemic, the measures used to ‘protect’ the nation have impacted significantly on children's health and well‐being^23^ and arguably will have a continued impact throughout many children's lives in terms of missed education and opportunities.^24^ Whilst there have been pledges from the UK government to invest in children's futures, through an education catch‐up fund, there is still little attempt to engage with children and young people's hopes and fears about the pandemic, including their views on the vaccination programme, or how schooling and educational settings should ‘look like’ and safely operate in the year(s) ahead.^25^

Whilst the UK's response to the pandemic has not been specifically tailored for children, it was clear that children in this study had a strong sense of the role that they could and should take. Their social responsibility in relation to their family, school and wider community came through clearly. Such findings have been echoed by Bray et al.^26^, ^27^ in their international survey of young children's health‐related knowledge during the COVID‐19 pandemic. Bray et al.^27^ reported children's strong sense of altruism, highlighting children's willingness to make sacrifices to their own lives to support others. Such contributions remain unacknowledged in the wider public sphere. Similarly, some children in our study reflected positively on the opportunities afforded by the pandemic and reported feeling proud to be part of a collective effort. In some ways, these inclusive narratives of belonging and feeling part of a wider national effort may perhaps reflect participants’ uptake of some of the early pandemic messaging that focused on a collective effort to support the NHS. They also provide further evidence of the children's positive contributions to the pandemic response and the insights that they can bring to the post‐COVID recovery period and beyond.

Despite the often adult‐centric nature of the public health discourse, participants had a clear sense of what they should be doing in relation to infection control. Similar findings have been reported by Bray et al.^26^, ^27^ However, our study also highlights some of the potential unintended consequences of children's successful uptake of the public health messaging. There was a clear sense of fear expressed by children for their families and friends, with a particular emphasis on death and dying. Although the children in this study had a tendency to reflect on the assets and positive aspects of their ‘new normal’, we should not underestimate the potential longer‐term mental health impacts posed both by the pandemic and from the public health discourse. As this study was undertaken during the first wave of the pandemic, lockdown was still very much a novel concept. It would be important to explore children's experiences and the impact on their lives from the ongoing and longer‐term COVID‐19 measures.

Yet, despite children's expressed fears and sadness, our findings point to the ways in which children have forged new narratives within the public health restrictions. Accounts of outdoor play, creative approaches to keeping in touch with their friends and the use of technologies as a form of ‘freedom’ in an otherwise restricted world reflect the ways in which these children continued to engage with their peers, albeit in different virtual contexts. Evidence of this kind points to children's tenacity and positivity, despite the significant disruption to their childhoods. Harnessing these positive narratives within future public health measures may help to counter some of the more negative aspects of lockdown and the broader impacts of the pandemic on young lives, crucially enabling children to feel more ‘in control’ at a time of considerable uncertainty.

As with all research, there are limitations to our findings. The research was carried out during the first wave of the COVID‐19 pandemic and thus reflects the state of the pandemic and the related responses during that time. We appreciate that it has been a continuously evolving situation and that public health discourses and responses may have shifted since we collected our data. Second, the children drawn on for this study clearly did not reflect a diverse group, especially those children who come from more marginalized and vulnerable circumstances and who are likely to experience more significant (adverse) impacts as a result of the pandemic. Second, the necessary use of technology for children to take in the study sometimes hindered the potential to develop rapport with children and also limited the potential to engage with the drawing method. Further, the presence and/or proximity of parents within some interviews could have impacted on some children's responses. We are reflecting on these and broader methodological and ethical issues of conducting online research with children during a pandemic in a forthcoming paper. Despite these shortcomings, the findings from this study highlight the importance of creating opportunities to harness children's perspectives and engage them in debates about contemporary global challenges that directly impact on their lives.

## Data Availability

The data that support the findings of this study are available from the corresponding author upon reasonable request.
